# Codes, Clues, and Clinical Skills: Building a Psychiatric Escape Room

**DOI:** 10.1192/bjo.2026.11325

**Published:** 2026-06-30

**Authors:** Jasleen Deol, Joanna Pegg

**Affiliations:** 1 Aston University, Birmingham, United Kingdom; 2 Black Country Healthcare NHS Foundation Trust, Dudley, United Kingdom

## Abstract

**Aims:**

Innovative teaching methods and gamification are increasingly sought in medical education to promote active learning and engagement amongst students. This interactive andnovel approach has gained popularity within medical education and allows students to consolidate their clinical knowledge and apply their skills to unfamiliar scenarios.Students are encouraged to think in a holistic manner, and this teaching intervention facilitates experiential learning.

The aim of this study was to design and deliver a psychiatry-themed escape room for Fourth year medical students at Bushey Fields Hospital. This is a novel approach to undergraduate psychiatric teaching, which focused on consolidating clinical knowledge regarding basic principles of psychiatry and encouraging collaborative problem-solving.

**Methods:**

A 45–60minute escape room was developed, centred around a fictional patient who was admitted to hospital after experiencing psychosis. Students were given a backpack with a padlock that contained clues and five tasks to solve. Working in groups of 3–5, students solved tasks centred around history taking, mental state examinations, assessing risk, physical health monitoring and contemplating management approaches.

A mixed method approach was undertaken, and pre- and post-session questionnaires (Likert scaled) assessed student-rated confidence regarding management of psychiatric situations, assessing risk, formulating differentials and management plans were distributed. Further qualitative data was gathered from students regarding their learning and experiences regarding the psychiatry-themed escape room.

**Results:**

Eighteen students participated over four escape room sessions. Post-session confidence scores increased across all measured domains, especially when undertaking risk assessments and knowledge of the Mental Health Act.

Qualitative data was thematically analysed and students reported that this was a useful opportunity to apply their skills and knowledge, using a simulated case.

**Conclusion:**

The results of this study suggest that this approach helped students to engage with psychiatric teaching in a novel and innovative manner. Students reported that they felt that consolidating their psychiatric knowledge using a simulated case was a safe environment to learn further.

Students reported a greater appreciation regarding the use of legal frameworks, history taking skills and undertaking mental state examinations. These are key considerations and skills used within psychiatry, which will lead to resident doctors being well equipped with key psychiatric principles, improvement in future practice and patient care.

This psychiatry-themed escape room will be offered at other sites within the Trust, and future work includes possible expansion to resident doctors early within their training based locally.

